# The origin of populations of *Arabidopsis thaliana *in China, based on the chloroplast DNA sequences

**DOI:** 10.1186/1471-2229-10-22

**Published:** 2010-02-08

**Authors:** Ping Yin, Juqing Kang, Fei He, Li-Jia Qu, Hongya Gu

**Affiliations:** 1 National Laboratory of Protein Engineering and Plant Genetic Engineering, Peking-Yale Joint Center for Plant Molecular Genetics and AgroBiotechnology, College of Life Sciences, Peking University, Beijing 100871, China; 2 National Plant Gene Research Center (Beijing), Beijing 100101, China

## Abstract

**Background:**

In the studies incorporating worldwide sampling of *A. thaliana *populations, the samples from East Asia, especially from China, were very scattered; and the studies focused on global patterns of cpDNA genetic variation among accessions of *A. thaliana *are very few. In this study, chloroplast DNA sequence variability was used to infer phylogenetic relationships among *Arabidopsis thaliana *accessions from around the world, with the emphasis on samples from China.

**Results:**

A data set comprising 77 accessions of *A. thaliana*, including 19 field-collected Chinese accessions together with three related species (*A. arenosa*, *A. suecica*, and *Olimarabidopsis cabulica*) as the out-group, was compiled. The analysis of the nucleotide sequences showed that the 77 accessions of *A. thaliana *were partitioned into two major differentiated haplotype classes (MDHCs). The estimated divergence time of the two MDHCs was about 0.39 mya. Forty-nine haplotypes were detected among the 77 accessions, which exhibited nucleotide diversity (π) of 0.00169. The Chinese populations along the Yangtze River were characterized by five haplotypes, and the two accessions collected from the middle range of the Altai Mountains in China shared six specific variable sites.

**Conclusions:**

The dimorphism in the chloroplast DNA could be due to founder effects during late Pleistocene glaciations and interglacial periods, although introgression cannot be ruled out. The Chinese populations along the Yangtze River may have dispersed eastwards to their present-day locations from the Himalayas. These populations originated from a common ancestor, and a rapid demographic expansion began approximately 90,000 years ago. Two accessions collected from the middle range of the Altai Mountains in China may have survived in a local refugium during late Pleistocene glaciations. The natural populations from China with specific genetic characteristics enriched the gene pools of global *A. thaliana *collections.

## Background

*Arabidopsis thaliana *(L.) Heynh is an annual weed belonging to the family Brassicaceae (Cruciferae). The species is native to Europe and Central Asia, but is now widely distributed in the Northern Hemisphere ranging from 68°N (northern Scandinavia) to Equator (mountains of Tanzania and Kenya) [1]. Many characteristics, from morphological traits to protein and DNA markers, have been used to evaluate natural genetic variation among populations, and to reconstruct an intraspecific phylogeny, for *A. thaliana *(for example, [2-9]). It has been found that many nuclear genes comprise two or more major differentiated haplotypes, generally referred to as allelic dimorphism [10-20]. Balancing selection or ancient population subdivision was often invoked to explain the pattern. The major mechanisms for balancing selection are heterozygote advantage, frequency-dependent selection, or environmental heterogeneity. It is well known that *A. thaliana *has an inbreeding mating system. The estimated outcrossing rate of the species is 1% or less [21]. It seems difficult to imagine that so many loci in *A. thaliana *have experienced balancing selection via heterozygote advantage [22]. Therefore, frequency-dependent selection and/or diversifying selection might be the driving forces for the dimorphism phenomenon, as in the case of pathogen resistance (*R*) genes [17,18,23]. It is not clear yet if the dimorphism also exists in the chloroplast genome.

The chloroplast genome of *A. thaliana *is a circular DNA composed of 154,478 bp with a pair of inverted repeats of 26,264 bp separated by small and large single-copy regions of 17,780 bp and 84,170 bp, respectively [24]. The uniparentally inherited chloroplast genome has been utilized in many studies in plant population and evolutionary genetics. However, studies focused on global patterns of cpDNA genetic variation among accessions of *A. thaliana *are scattered. In an investigation on the maternal origins of *A. suecica*, 12 cpDNA regions were sequenced for 25 *A. thaliana *accessions, which were mainly collected from Scandinavia [25]. These authors found considerable variation existed among the non-coding single-copy sequences in the chloroplast genome of *A. thaliana*. In another study, the *trnL*-*trnF *cpDNA intergenic spacer region of 475 individuals from 167 *A. thaliana *populations in its native range was sequenced and 16 haplotypes were identified [8]. Based on the chloroplast and nuclear DNA sequence data, Beck *et al *proposed the Caucasian area as the possible ancestral area of *A. thaliana*, and suggested four possibilities for the origin of East Asian populations. They also found that the maternal components of *A. suecica *shared a high similarity to those in the Asian metapopulation of *A. thaliana*, especially to those from China [8].

In the studies incorporating worldwide sampling of *A. thaliana *populations, the samples from East Asia were very scattered. He *et al *conducted a study on the genetic diversity of 19 natural *Arabidopsis thaliana *populations in China based on ISSR and RAPD makers, and found that about 42-45% of the total genetic variation existed within populations and there was a significant correlation between geographic distance and genetic distance [7]. However, the phylogenetic relationships of Chinese populations with those distributed in other regions of the world, and the history of population dispersal in this region, are not clear.

The goals of the present survey are: (1) to examine global patterns of cpDNA genetic variation in *A. thaliana*; (2) to infer phylogenetic relationships among *A. thaliana *accessions from all over the world based on cpDNA sequence data, with particular focus on Chinese populations; and (3) to discuss the possible origin(s) of the Chinese populations. It was found in this study that dimorphism did exist in the chloroplast genome of *A. thaliana*; the 77 accessions studied were grouped into two major clusters; and the Chinese populations might have two independent origins.

## Results

### Nucleotide variation in the chloroplast DNA sequences

**Figure 1:**
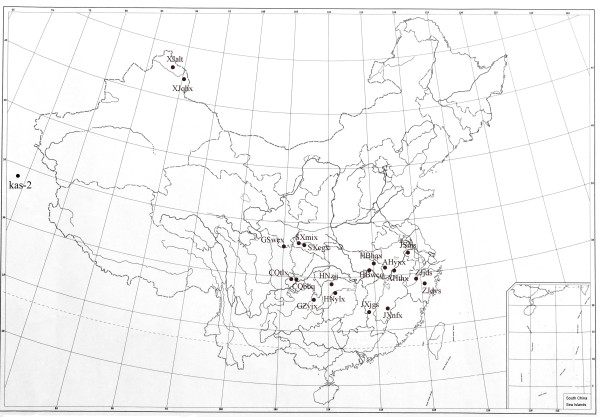
**Distribution map of the 19 accessions of *Arabidopsis thaliana *from China and one from India (Kas-2)**. Solid circles indicate the locations where samples were collected.

Seventy-seven *A. thaliana *accessions were used in the survey, among them 19 accessions were field collected in China (Table 1). All sampling locations in China were separated by at least 50 km, with most of the locations separated by more than 300 km (Figure 1, Table 2). No cp DNA polymorphism was detected within either accession AHyxx or Abd-0, therefore only one individual was chosen for each accession for DNA sequence analysis. About 10600 nucleotides from the chloroplast genome were amplified and sequenced for each accession, of which 8750 nucleotides of non-coding fragments were retained for analysis.

**Table 1:** List of the *A. thaliana *accessions used in this study

	*Name*	Accession no. *	*Geographic Origin*		*Name*	Accession no. *	*Geographic Origin*
1	9481	N22458	Kazakhstan	39	KZ10	N22442	Kazakhstan
2	Aa-0	N934	Germany	40	La-0	N1298	Poland
3	Abd-0	CS932	UK	41	Lc-0	CS6769	Scotland
4	Ag-0	N936	France	42	Lip-0	N1336	Poland
5	Al-0	N940	Denmark	43	Mr-0	N1372	Italy
6	Alc-0	N1656	Spain	44	Ms-0	N905	Russia
7	Ang-0	N948	Belgium	45	Mt-0	N1380	Libya
8	Anholt-1	CS22313	Germany	46	N1	N22479	Russia
9	Ba-1	N952	UK	47	Ost-0	N1430	Sweden
10	Berkeley	N8068	USA	48	Per-2	N1448	Russia
11	BG1	N22341	USA	49	Pi-0	N1454	Austria
12	Bl-1	CS6615	Italy	50	Pog-0	N1476	Canada
13	Blh-1	N1030	Czech Republic	51	Rubezhnoe-1	N927	Ukraine
14	Bs-1	N996	Switzerland	52	Sei-0	N1504	Italy
15	Bur-0	N1028	Ireland	53	Sorbo	N931	Tajikistan
16	Bus-0	N1056	Norway	54	Ta-0	N1548	Czech Republic
17	Cal-0	N1062	UK	55	Te-0	CS6918	Finland
18	Can-0	N1064	Spain	56	Tsu-0	N1564	Japan
19	Cha-0	N1068	Switzerland	57	Wassilewskija	N915	Russia
20	Chi-0	N1072	Russia	58	Wil-1	N1594	Lithuania
21	Col-0	N1092	USA	59	XJalt	PKU101	China
22	Ct-1	N1094	Italy	60	XJqhx	PKU102	China
23	Cvi-0	N1096	Cape Verde Island	61	CQbbq	PKU304	China
24	Eil-0	N1132	Germany	62	CQtlx	PKU305	China
25	Es-0	N1144	Finland	63	GSwex	PKU306	China
26	Est-0	N1148	Previous USSR	64	GZyjx	PKU603	China
27	FM10	N22391	USA	65	HNylx	PKU602	China
28	For-1	N1164	UK	66	HNzjj	PKU601	China
29	Gr-3	N1202	Austria	67	SXcgx	PKU308	China
30	Gy-0	N1216	France	68	SXmix	PKU307	China
31	Hi-0	N1226	Netherlands	69	AHthx	PKU218	China
32	Hirokazu	N3963	Japan	70	AHyxx	PKU219	China
	Tsukaya			71	HBhax	PKU309	China
33	HR14	N22213	UK	72	HBwcq	PKU303	China
34	HS10	N22354	USA	73	JSnjs	PKU301	China
35	Ita-0	CS1244	Morocco	74	JXjgs	PKU210	China
36	Kas-1	CS903	India	75	JXnfx	PKU207	China
37	Kas-2	CS1264	India	76	ZJdys	PKU205	China
38	Kn-0	N1286	Lithuania	77	ZJjds	PKU201	China

*Accession no. begun with 'N' were obtained from the Nottingham Arabidopsis Stock Center (NASC); with 'CS' were obtained from the Arabidopsis Biological Resource Center (ABRC); with "PKU" were field collected in China and their detailed information is listed in Table 2.

**Table 2:** Geographic information for the 19 accessions collected from China

*Name*	*Location*	*Latitude and longitude*	*Altitude (m)*
XJalt	Xinjiang, Aletaishi	47°46' 72" N 88°20' 64" E	830
XJqhx	Xinjiang, Qinghexian	46°48' 72" N 90°20' 39" E	1400
CQbbq	Chongqing, Beibeiqu	29°47' 41" N 106°28' 64" E	184
CQtlx	Chongqing, Tongliangxian	29°49' 40" N 106°03' 38" E	263
GSwex	Gansu, Wenxian	32°43' 30" N 105°07' 21" E	650
GZyjx	Guizhou, Yinjiangxian	27°56' 64" N 108°36' 49" E	800
HNylx	Hunan, Yuanlingxian	28°31' 23" N 110°43' 13" E	200
HNzjj	Hunan, Zhangjiajie	29°24' 45" N 110°26' 33" E	500
SXcgx	Shanxi, Chengguxian	32°55' 93" N 107°12' 65" E	607
SXmix	Shanxi, Mianxian	33°08' 82" N 106°44' 72" E	532
AHthx	Anhui, Taihuxian	30°27' 80" N 117°17' 79" E	120
AHyxx	Anhui, Yuexixian	30°42' 89" N 116°15' 33" E	600--800
HBhax	Hubei, Honganxian	31°16' 70" N 115°01' 11" E	100
HBwcq	Hubei, Wuchangqu	30°31' 32" N 114°28' 16" E	70
JSnjs	Jiangsu, Nanjingshi	32°03' 12" N 118°49' 93" E	60
JXjgs	Jiangxi, Jinggangshan	26°44' 78" N 114°17' 98" E	390
JXnfx	Jiangxi, Nanfengxian	26°59' 18" N 116°14' 51" E	360
ZJdys	Zhejiang, Dongyangshi	29°05' 02" N 120°25' 65" E	290
ZJjds	Zhejiang, Jiandeshi	29°32' 13" N 119°29' 61" E	100

**Figure 2:**
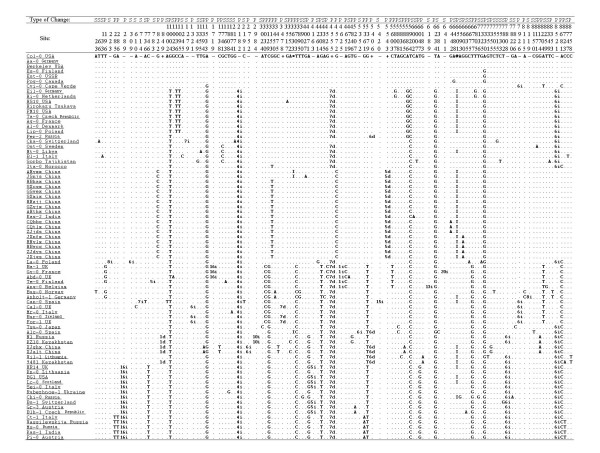
**The 123 polymorphic variations in the combined data matrix**. In the "Type of Change", S = singleton site; P = parsimony informative site. The numbers in the "Site" denote the nucleotide sites at which the variations occurred in the combined data matrix. In the first row of the data matrix, the capital letters indicate the nucleotides in Col-0, a minus sign (-) indicates a deletion whereas a plus sign (+) indicates an insertion in certain accession(s) relative to Col-0, * and @ indicate the sites which two small fragment inversions were located. In the data matrix, # d = deletion of # nt; # i = insertion of # nt; I = inversion relative to the first sequence (Col-0), and a dot indicates the same nucleotide as in the first sequence (Col-0).

The combined data matrix contained 149 variable nucleotide sites. Among them, 21 were mononucleotide repeat polymorphisms, one was a dinucleotide repeat polymorphism, and four were complicated length variations. These 26 length polymorphisms were excluded in the analyses. The other 123 polymorphic variations comprised 95 single nucleotide polymorphisms (SNPs), 26 insertion/deletion events (indels) and two small fragment inversions. Only one site exhibited three-base polymorphism and the other 122 sites showed two-base polymorphism (Figure 2).

**Figure 3:**
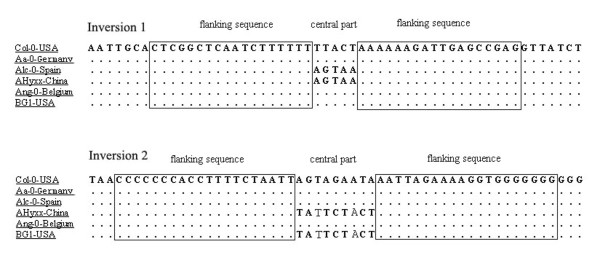
**Two inversions found in cp-genome of *A. thaliana***. The dots denote the same nucleotides as in Col-0. The two franking sequences are reversely complemented to each other but maintain invariable in all accessions studied (except for Pog-0) whereas the central part may mutate to its reverse complementary sequence.

The two small fragmental inversions were located at sites 3633-3637 (Inversion 1) and sites 6501-6509 (Inversion 2). The characteristics of this kind of reversion were: (1) a central region of 5 nt (TTACT in the Inversion 1 of Col-0) or 9 nt (AGTAGAATA in the Inversion 2 of Col-0), which could mutate to its reverse complement sequence (AGTAA and TATTCTACT, respectively); and (2) two flanking sequences of 18 nt (Inversion 1) or 20 nt (Inversion 2), respectively (Figure 3). The two flanking sequences could be reversely complemented to each other. It is most likely that each of these two small fragmental inversions could be generated by only one or very few mutation event(s), but it resulted in multiple SNPs.

Nucleotide diversity (π) for the entire sequenced regions was 0.00169, but ranged from 0.00010 for the *ycf3-trnS *intergenic spacer (primer pair 4) to 0.01053 for *psaJ-rpl33 *(primer pair 9) (Table 3).

**Table 3:** Nucleotide diversity (π) and the results of neutral mutation hypothesis tests for the 11 fragments data sets

*Primer No.*	*π*	*Tajima's* *D*	Fu and Li's D*	Fu and Li's F*	*Fu and Li's D*	*Fu and Li's F*
1	0.0017	-1.4447 NS	-1.6261 NS	-1.8451 NS	0.0183 NS	-0.4667 NS
2	0.0014	-1.1220 NS	-2.6973*	-2.5490*	-2.4816*	-2.3338*
3	0.0010	-1.4093 NS	0.0316 NS	-0.5085 NS	1.0701 NS	0.3136 NS
4	0.0001	-1.8133*	-3.7112**	-3.6475**	-3.7960**	-3.7259**
5	0.0027	-0.2615 NS	-0.4679 NS	-0.4713 NS	-0.4989 NS	-0.4973 NS
6	0.0021	-0.8466 NS	0.0413 NS	-0.3111 NS	0.0244 NS	-0.3331 NS
7	0.0017	-0.6747 NS	-1.0450 NS	-1.0878 NS	-1.0991 NS	-1.1344 NS
8	0.0015	-1.1267 NS	-0.3436 NS	-0.7422 NS	-0.3861 NS	-0.7901 NS
9	0.0105	1.26884 NS	-0.4650 NS	0.1805 NS	-0.2106 NS	0.3956 NS
10	0.0003	-2.18989**	-3.9879**	-3.9933**	-4.1744**	-4.1512**
11	0.0012	-1.4249 NS	-1.8752 NS	-2.0389NS^a^	-1.1622 NS	-1.4037 NS
Comb.	0.0017	-1.1723 NS	-2.3669*	-2.2576NS^a^	-2.0618NS^a^	-2.0080NS^a^

NS = Not significant and P > 0.10

NS^a ^= Not significant but 0.10 > P > 0.05

* P < 0.05

** P < 0.02

Less frequent nucleotide polymorphisms (such as singleton or doubleton) were in excess for the sequenced regions. Singletons were found at a very high frequency: 44 among the 95 SNPs and 10 among the 26 indels were singletons (Figure 2). The excess of low-frequency polymorphisms resulted in negative Tajima's D, Fu and Li's D* and F* values for most of the sequenced segments; for example, 10 out of 11 Tajima's D values, nine out of 11 Fu and Li's D* values and 10 out of 11 Fu and Li's F* values were negative (Table 3). The values for the combined data matrix were -1.17234 (Tajima's D, *P *> 0.10), -2.36692 (Fu and Li's D*, *P *< 0.05) and -2.25760 (Fu and Li's F*, 0.10 > *P *> 0.05, critical). When Fu and Li's D and F tests were conducted using the *A. arenosa *ortholog as the reference sequence, similar results were obtained: eight out of 11 Fu and Li's D values and nine out of 11 Fu and Li's F values were negative and for the combined data matrix; both the values were negative (-2.06178 and -2.00798, respectively, 0.10 > *P *> 0.05; Table 3).

In total, we identified three types of nucleotide variations among the aligned sequences: SNPs, length polymorphisms (including indels), and two small fragmental inversions.

### Phylogenetic relationships among the accessions

**Figure 4:**
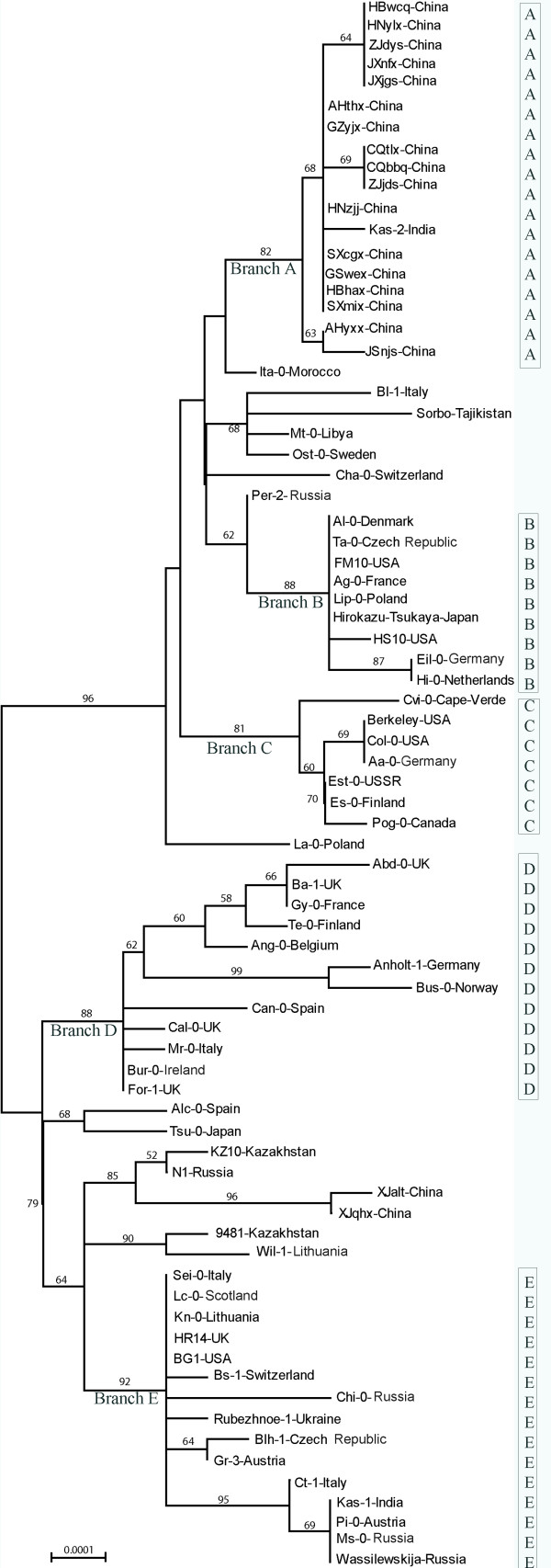
**NJ tree based on the combined data matrix**. Bar at the left bottom indicates scale value. Numbers at nodes indicate bootstrap values. All nodes with <50% bootstrap support are collapsed.

**Figure 5:**
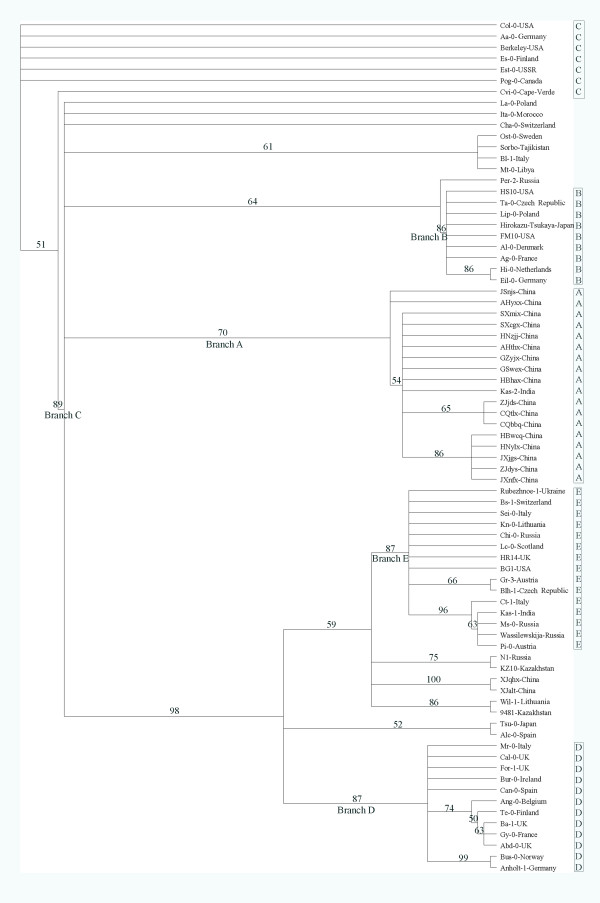
**MP tree inferred from the combined data matrix**. The numbers at nodes indicate bootstrap values. All nodes with <50% bootstrap support are collapsed.

Because the single base changes in the two short inverted regions were not independent events, they were excluded from the phylogenetic analysis. Two distinct clusters with high bootstrap values were retrieved in the NJ tree. One cluster included 42 accessions and the other included 35 accessions (Figure 4). Although the topology of the MP tree differed to that of the NJ tree, one branch with 35 accessions corresponded to one of the two clusters in the NJ tree (Figure 5). In general, no significant correlation was detected between geographical origins and clusterings in the phylogenetic trees. Accessions from the same country, such as four accessions from Italy (Bl-1, Ct-1, Mr-0 and Sei-0) and five accessions from USA (Berkeley, BG1, Col-0, FM10 and HS10) failed to cluster together, but were scattered on different branches. This lack of phylogeographic structure conforms to the hypothesis of a rapid recent expansion of the species with strong involvement of human-mediated migrations [1].

Although there was incongruence between the two phylogenies, the topological relationship was relatively stable among a large number of accessions. We identified four stable branches in both trees (A, B, D, and E in Figures 4 and 5). The only major difference was in branch C. It was placed within one of the two clusters in the NJ tree, but formed a deep polytonous branch in the MP tree, containing the same accessions except Cvi-0, an accession from Cape Verde Island. The branches A-E comprised 61 out of the 77 accessions.

### Discrimination of two major differentiated haplotypes among *A. thaliana *accessions

**Figure 6:**
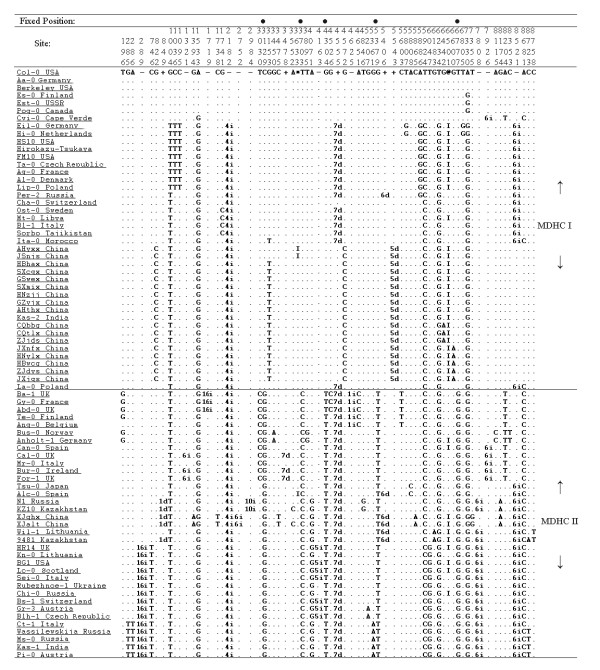
**The 77 sequences were structured into two major differentiated haplotype classes**. The solid circles in the first line denote the five fixed nucleotide sites where the two MDHCs differ.

When only the parsimony-informative sites were considered, the nucleotide variation of the 77 accessions was structured into two major different haplotype classes (MDHCs, Figure 6). The MDHC-I and MDHC-II classes were composed of 42 and 35 accessions, respectively, and they corresponded well to the two clusters in the NJ tree. The MDHC-I and MDHC-II classes differed at five nucleotide sites (C to G at site 3129, T to C at site 3703, G to T at site 4304, G to T at site 5379, and T to G at site 6777; Figure 2), and these sites were within a fragment about 20 kb long from *trnL *to *rpl33 *in the chloroplast genome.

**Figure 7:**
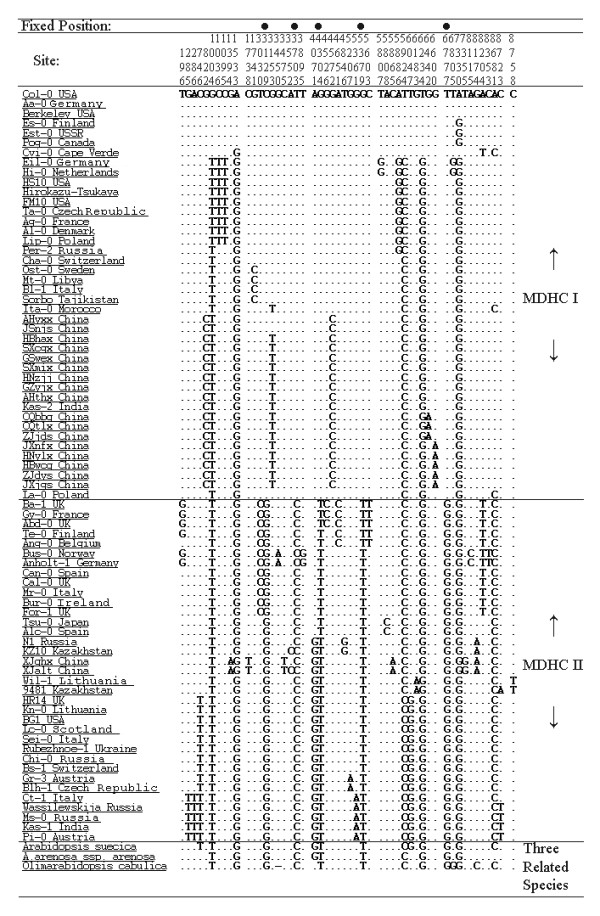
**Selected parsimony-informative SNP sites in the 77 accessions of *A. thaliana *and three related species**. The solid circles indicate the sites where the three related species were identical to the MDHC-II.

For interspecific comparison, the homologous sequences of three related species, *Olimarabidopsis cabulica*, *A. arenosa *and *A. suecica*, were aligned with the 77 *A. thaliana *accessions. They were identical to those in MDHC-II of *A. thaliana *at all five nucleotide sites where the two MDHCs could be distinguished from each other (Figure 7).

**Figure 8:**
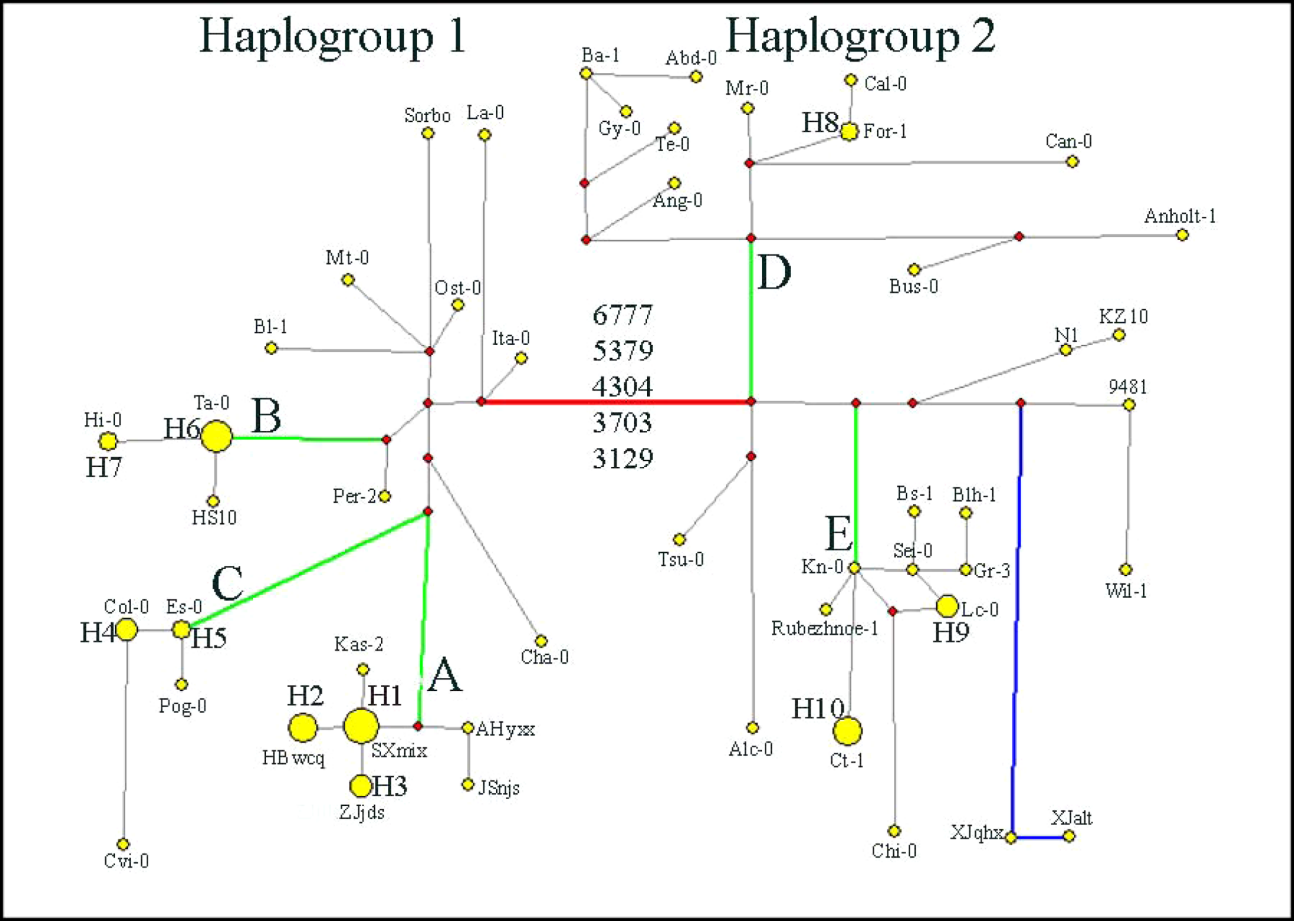
**Haplotype network**. The red circles indicate median vectors and yellow circles indicate haplotypes. The areas of the yellow circles are proportion to the number of accessions in each haplotype and the length of the lines between circle midpoints are proportion to the differences between haplotypes. The haplotype is denoted by the accession name if there is only one accession in the haplotype, otherwise the haplotype is denoted by H1~H10. The Arabic numerals denote the five sites where the two haplogroups differ. The five green lines and the capital letters (A, B, C, D, E) beside the lines denote five clusters corresponding to the five branches (Branch A, B, C, D, E) in the NJ and MP trees. H1: AHthx, GSwex, GZyjx, HBhax, HNzjj, SXcgx, SXmix; H2: HBwcq, HNylx, JXjgs, JXnfx, ZJdys; H3: CQbbq, CQtlx, ZJjds; H4: Aa-0, Berkeley, Col-0; H5: Es-0, Est-0; H6: Ag-0, Al-0, FM10, Hirokazu Tsukaya, Lip-0, Ta-0; H7: Eil-0, Hi-0; H8: Bur-0, For-1; H9: BG1, HR14, Lc-0; H10: Ct-1, Kas-1, Ms-0, Pi-0, Wassilewskija.

All the sites except the inverted length variants were used to form a binary data set for haplotype network analysis. Forty-nine haplotypes were identified in the 77 accessions of *A. thaliana*. The 49 haplotypes were also bifurcated to form two haplogroups (Figure 8). Haplogroup 1 (21 haplotypes) and Haplogroup 2 (28 haplotypes) differed at the same five sites (3129, 3703, 4304, 5379 and 6777) where MDHC-I and -II differed, and the accessions in Haplogroup 1 and Haplogroup 2 were identical to those in MDHC-I and -II, respectively.

### Estimated divergence time for MDHC-I and MDHC-II, and demographic expansion of a monophyletic group of accessions in Asia

The K value between *A. arenosa *and 77 *A. thaliana *accessions was 0.0280 ± 0.0037. Using Equation 1, the substitution rate per nucleotide site per year for the sequenced chloroplast regions was 2.8 × 10^-9^. The K value between MDHC-I and MDHC-II was 0.0022 ± 0.0005. Therefore, the estimated divergence time for MDHC-I and MDHC-II was estimated (using Equation 2) to be about 0.39 ± 0.09 mya.

Although no significant correlation was detected between geographic origin and genetic distance, the 17 accessions collected along the Yangtze River, China, were always clustered together with Kas-2, an accession from Kashmir (74°E, 34°N) in both NJ and MP trees (Figures 4 and 5). In the network analysis, they congregated closely and formed a distinct cluster (Cluster A) in Haplogroup 1 (Figure 8). The level of nucleotide polymorphism of the 18 accessions was very low (π = 0.00030), only six haplotypes (*h*) were detected (five specific in the 17 accessions along the Yangtze River) and haplotype diversity (*Hd*) was 0.778. In comparison, the values of π, *h *and *Hd *for the 77 accessions were 0.00169, 49 and 0.977, respectively.

To test the model of demographic population growth in the region from which the 18 accessions were sampled, especially for the 17 accessions along the Yangtze River, a mismatch distribution analysis was conducted. Each small fragment inversion was treated as a SNP in the analysis. The SSD between the observed and expected mismatch distribution was 0.093 (*P *= 0.062) and HRag was 0.300 (*P *= 0.022). There was an only marginally significant difference in SSD between the observed and the predicted pairwise difference distribution under the sudden-expansion model. This result provided evidence of rapid population expansion along the Yangtze River. The average τ-value was 4.521 (95% confidence intervals: 0.684~8.197). The initial time when the populations expanded along the Yangtze River were calculated using Equation 4 to obtain *u *(2.52 × 10^-5^), and then using Equation 3 to obtain *t *(0.897 × 10^5^). Therefore, the initial time of expansion was estimated to be about 90,000 years ago.

## Discussion

### The level and pattern of nucleotide variation in the sequenced chloroplast regions

The *π *value of the sequenced chloroplast regions among global samples of *A. thaliana *accessions was 0.00169, which is about one-quarter of that of the mean nucleotide diversity of the nuclear genes in *A. thaliana *[1], but double that in another study by Sall *et al *[25], in which 12 non-coding single-copy cpDNA regions were sequenced for 25 *A. thaliana *accessions (*π *= 0.00061). The differences may be due to the different sampling strategies. The 25 *A. thaliana *accessions in the latter study were mainly collected from Scandinavia, whereas the 77 accessions in the present study were sampled worldwide. For a highly self-fertilizing species, geographical structure may play an important role on a smaller scale in the level of polymorphism, at least for the uniparentally inherited chloroplast genome. For example, the π value reduced to 0.00030 if only 18 accessions in branch A (Kas-2 and 17 accessions along the Yangtze River) were considered.

Inversions in the chloroplast genome exist in monocotyledonous plants and the Asteraceae. The length of these inversions range from 0.5 to 28 kb, and all have phylogenetic implications [26,27]. The length of the inversions found in the present study were much shorter, only about 18-20 bp. The accessions with inversions were found mostly scattered on branches B, D, E in the NJ and MP trees. The exception is in branch A, where all accessions had inversion 2. The mechanism responsible for these inversions is not known, but they might have originated several times during the population expansion process. Therefore, it is advisable not to consider them for phylogenetic analysis.

### Dimorphism in the chloroplast DNA of *A. thaliana*

Two significantly differentiated haplotype classes could be identified in the sequenced chloroplast DNA regions, just as in the allelic dimorphism found in some nuclear DNA sequences of *A. thaliana*. At least three different interpretations have been proposed to explain the nuclear dimorphism phenomenon. First, balanced polymorphisms were usually the mutations maintained in populations by natural selection through heterozygotic advantage [17,28]. The chloroplast genome is maternally inherited in *A. thaliana*, and the DNA regions selected for analysis in this study are intergenic regions. Therefore, the dimorphism found in the chloroplast may not be caused by balancing selection via heterozygotic advantage. Furthermore, in our investigation, the value of the Tajima's D-value was negative. A negative Tajima's D value is a general feature of the *Arabidopsis thaliana *genome [29], and is correlated to demographic factors, such as population growth [30], rather than non-neutral forces such as selection [8].

A second explanation for the nuclear dimorphism is that introgression might result in the allelic dimorphism. Chloroplast DNA introgression has been widely reported [e.g., [31,32]]. In this study, we found that two related species, *Olimarabidopsis cabulica *and *A. arenosa*, had all five identical nucleotide site variations with MDHC-II of *A. thaliana*, which were the 'markers' to separate MDHC-II from MDHC-I. However, the K values between *O. cabulica *and the 77 accessions of *A. thaliana*, and between *A. arenosa *and the 77 *A. thaliana *accessions, are 0.0395 and 0.0280, respectively, whereas that between the two MDHCs of *A. thaliana *is only 0.0022. The interspecific genetic distance is at least one order of magnitude higher than intraspecific genetic distances. The estimated divergence time between *O. cabulica *and *A. thaliana *is about 10~14 mya, and that between *A. arenosa *and *A. thaliana *is about 3.0~5.8 mya [33]. The genetic distances based on cpDNA between these species pairs correlate to their nuclear gene-based estimated divergent time. These results indicated that the dimorphism in cpDNA found in this study was not the result of recent introgressive hybridization events, but we cannot rule out the possibility that the dimorphism might be the result of ancient introgression events. Hybridization between *A. thaliana *and its closely related species does occur in nature. For example, several studies confirmed the allotetraploid species, *A. suecica*, resulted from a hybridization event between *A. thaliana *and *A. arenosa *about 10,000 to 50,000 years ago [e.g., [25,34,35]].

The third explanation for genetic dimorphism is demographic factors, such as founder effects. Being a small annual weed, *A. thaliana *is a poor competitor in dense vegetation whereas the highly self-fertilizing characteristic makes it capable of founding a population even from a single seed. As a result, this species has a tendency for rapid colonization and extinction cycles [1,7,36]. Founder effects might have occurred repeatedly in the evolutionary history of *A. thaliana*. The founder event(s) could enable some rare alleles to spread into additional populations when the founder population expanded rapidly if the unoccupied ecological niches were favourable. The divergence time between MDHC-I and MDHC-II was estimated to be about 0.36 mya based on our cpDNA data. This is earlier than the estimated time of demographic expansion during the Eemian interglacial (about 0.122 mya; [8]). Therefore, another possible explanation for the cpDNA dimorphism might be a founder effect followed by limited gene flow during late Pleistocene glaciations and interglacial periods.

As the accessions in MDHC-II share five specific variable sites with *A. arenosa*, the chloroplast genomes in MDHC-II might represent more ancient types than those in MDHC-I. It is also supported by the fact that more haplotypes are found in MDHC-II (28) than in MDHC-I (21).

### Origin of Chinese populations

The 26 accessions of *A. thaliana *from Asia included in this study are scattered compared to those collected from Europe. Six of them belong to MDHC-II and 20 belong to MDHC-I. Of the MDHC-II group, two collections from China (XJalt and XJqhx) and two from Kazakhstan (9481 and Kz10) are within or very close to the Altai Mountains. Although these four accessions were not clustered in the same clade in the phylogenies, the two accessions from China were always on the same branch. One of the Kazakhstan accessions (Kz10) was clustered with XJalt and XJqhx together with a Russian accession (N1, from Europe) in the NJ tree (Figure 4). XJalt and XJqhx are unique in that they share six specific variable sites (Figure 2), the most number of specific variable sites in this study. The provenances of these two accessions are about 115 km apart and located in the middle of the Altai Mountains range. Based on the cpDNA data, the populations on the Altai Mountain range may have dispersed there during one of the late Pleistocene glaciations, and some local habitats along the southern slopes of the Altai Mountains might have served as refugia. In contrast to some refugia in Europe, where *A. thaliana *populations had contributed the postglacial colonization of western and northern Europe [9], some populations in the Asian refugia, such as XJalt and XJqhx, became relatively isolated genetically from other populations after glaciers retreated. Therefore, some fixed mutations were accumulated specifically in these populations. It is also noticed by Beck et al [8] that "differentiation is particularly strong between the Central Asian refugia and all others, suggesting that either small historical population sizes in Central Asia, relatively limited gene flow between Central Asia and other areas, or a combination of the two have produced relatively strong genetic drift in this region". The other two Asian accessions in the MDHC-II group are from Japan (Tsu-0) and India (Kas-1). Although Tsu-0 was clustered with Alc-0 from Spain, the bootstrap support is poor in both NJ and MP trees (68% and 52%, respectively). Tsu-0 has two specific variable sites, but only shares one specific site with Alc-0. Its phylogenetic relationships with other accessions are unclear and more samples are needed to clarify its origin. Kas-1 from India shares four specific variable sites with four accessions from Europe (Figure 2); this accession will be discussed later.

In MDHC-I, 17 accessions collected along the Yangtze River, China, were clustered together with an accession from India (Kas-2). All data suggested that the chloroplast genomes of these 18 accessions originated from a single common ancestor. The initial time of expansion of the populations along the Yangtze River was estimated to be about 90,000 years ago based on the cpDNA sequences. At least we can rule out the possibility that these populations were introduced by recent human activities, like the populations found in the USA. Both highlands of the western Himalayas and Caucasus have been proposed as possible ancestral areas of *A. thaliana *[8,37]. The populations along the Yangtze River could have dispersed eastwards from the ancestral area to the present-day locations via the Himalayas or Kunlun Mountains, which have an east-west trend. Kas-2 was collected in Kashmir, India (74°E, 34°N), on the southern slopes of the Himalayas. It shares a most frequent haplotype with seven Chinese accessions, but it can be distinguished from them only by one specific variable site (Figure 2). There are at least two explanations for the observed distribution pattern. The first is that Kas-2 represents the type that is most similar to the ancestor of the Yangtze River populations, and the expansion of the latter was immediately or very shortly after the dispersal event. However, in the phylogenies, Kas-2 is not basal among the 18 accessions. The second explanation is that Kas-2 might have been introduced to Kashmir from China. It is uncertain which explanation is more likely. More samples from the western part of the Himalayas and Kunlun Mountains are definitely needed for identification of the possible ancestral haplotype of the Yangtze River populations.

The other three accessions in MDHC-I are H Tsukaya from Japan, Sorbo from Tajikistan, and Kas-1 from India. The former shares one haplotype with four accessions from Europe and one accession from the USA. It is apparent that it represents a recent introduction to Japan, possibly by human activity. The accession from Tajikistan has a specific haplotype that is characterized by four unique SNPs (Figure 2), and it shares a haplotype with one accession from Sweden, Italy and Libya, respectively. Kas-1 shares the exact haplotype with two accessions from Russia (Wassilewskija, Ms-0) and one from Austria (Pi-0). Although Kas-1 and Kas-2 were collected at the same locality (74°E, 34°N), as stated on the ABRC seed stock centre website, their genetic distinctness has been noted previously [38]. Re-sampling from their original locality might help in clarifying the confusion.

### The possible maternal parent of *Arabidopsis suecica*

*Arabidopsis suecica *is an allotetraploid species whose maternal parent is *A. thaliana*. The species originated more than 20,000 years ago from an allotetraploid hybrid between *A. thaliana *and *A. arenosa *[25,35]. Several studies indicate that the ancestral area of *A. suecica *is in Europe [e.g., [33]]. However, a recent study by Beck *et al *[8] revealed a genetic similarity between *A. suecica *and Chinese accessions of *A. thaliana*. In the present study, the cpDNA sequences of *A. suecica *were more similar to those of some European accessions, especially Chi-0 from Russia (34°E, 54°N). Based on the comparison of cpDNA in this study, it is most likely that the maternal parent of *A. suecica *was from Europe.

## Conclusions

Elucidating the dispersal of *A. thaliana *within Asia is a very complicated issue. Temperature fluctuations during glaciations and interglacial periods in the late Pleistocene, the complex mountain ranges in the Central and Central-East parts of Asia, and human activities have all contributed to the present-day distribution patterns. It is clear from this study that some populations in East Asia, such as those along the Yangtze River, were dispersed there along the Himalaya or Kunlun Mountain ranges and underwent rapid expansion about 90,000 years ago. Altai Mountains may have provided refugia for *A. thaliana *during Pleistocene glaciations. However, more samples from the Altai, Kunlun, and western part of the Himalaya mountain ranges are needed to elucidate more fully the dispersal history of *A. thaliana *populations in Asia.

## Methods

### Plants material and DNA extraction

Seventy-seven *A. thaliana *accessions were used in the survey, seeds of 50 accessions were obtained from the Nottingham *Arabidopsis *Stock Centre (NASC; University of Nottingham), eight accessions from the *Arabidopsis *Biological Resource Center (ABRC; Ohio State University) and 19 accessions were field collected in China (Table 1). All sampling locations in China were separated by at least 50 km, with most of the locations separated by more than 300 km (Figure 1, Table 2). The nomenclature of the Chinese accessions follows that of He *et al*. [7]. Two taxa closely related to *A. thaliana*, i.e., *A. arenosa *and *A. suecica*, and one taxon closely related to the genus *Arabidopsis*, i.e., *Olimarabidopsis cabulica*, were used as out-groups. Seeds of selected plants were sterilized and germinated on MS solid agar medium, and then transplanted into soil, as described previously [7]. The seedlings were grown in a controlled environment at 22°C with a photoperiod of 16 h light/8 h dark. About 1 g of green leaf material was harvested from a single plant for each accession and used for total cellular DNA extraction using the CTAB method [39].

### PCR amplification, DNA sequencing, and sequence alignment

Intron or intergenic regions were selected for PCR amplification in order to obtain maximum phylogenetic information. Eleven pairs of PCR primers were designed based on the Col-0 chloroplast genome sequences (GenBank accession no. AP000423) using the software Primer Premier [40]. All 11 pairs of primers are positioned in protein- or RNA-coding gene regions flanking the target fragments, and the length of the fragments ranged from 570 nt to 1496 nt (Table 4). The amplified products were purified with the PCR Purification and Gel Extraction Kit (V-gene Biotechnology), and sequenced with an ABI 377 Automated DNA Sequencer (Perkin-Elmer). To ensure the accuracy of the nucleotide sequences, the sequences on both strands were determined, all polymorphisms were visually confirmed, and for ambiguous polymorphisms PCR amplification and sequencing were repeated.

**Table 4:** Information about eleven pairs of primers for PCR amplification and the length of their products

*Primer No.*	*Intergenic region*	Site* (nt)	*Primer sequence*	*GenBank Acc No*
1	trnR-atpA	9662-9937	5'-GGATAGGACATAGGTCTTCTAA-3' 5'-CACAGTGGAAGAACAGATAATG-3'	GU293237-GU293316

2	rpoB-trnC	26330-27372	5'-CCCTTCAAATTGTATCTGATTAAA-3' 5'-GATTTGAACTGGGGAAAAAGGATT-3'	GU293317-GU293396

3	trnG-trnfM-rps14	36561-36937	5'-GCGGATATAGTCGAATGGTAAA-3' 5'-GTAAGATTCCGTCGCTAAGTGA-3'	GU293397-GU293476

4	ycf3-trnS	43752-44826	5'-CGCATAGCTTCATAATAATTCTGT-3' 5'-TCTACATAACAGTTCCAATGTTAC-3'	GU293477-GU293556

5	trnL-trnF	47491-48174	5'-TCCTCTGCTCTACCAACTGA-3' 5'-AAATCGTGAGGGTTCAAGTC-3'	GU293557-GU293636

6	rbcL-accD	56398-57074	5'-CTAGCTGCTGCTTGTGAAGTATGG-3' 5'-TAAAATTGAACCACGATTTTTCCA-3'	GU293637-GU293716

7	accD-psaI	58542-59246	5'-CAATTGCCGGAAAGACTAGG-3' 5'-GTTCACAAGCGGCTGAATCT-3'	GU293717-GU293796

8	psbE-ORF31	64323-65711	5'-TATCGAATACTGGTAATAATATCA-3' 5'-ATAGTTAAAGCTGCTAGTAGAAAA-3'	GU293797-GU293876

9	psaJ-rpl33	67064-67487	5'-CTAGGGGTGTTATGCCGATT-3' 5'-GTTCGGTTCGTTAGCAGGTT-3'	GU293877-GU293956

10	rpl20-clpP	68866-69909	5'-CATGGAACGGGATGTTTTTA-3' 5'-GTTCTACGCCTCCGAGCTAT-3'	GU293957-GU294036

11	Intron in rpl16	81588-82643	5'-TCCTCGATGTTGTTTACGAAATCT-3' 5'-TCGAACTATTTATGGGGTTTTAGG-3'	GU294037-GU294116

* The Col-0 chloroplast genome sequence was used as reference (GenBank Accession: AP000423).

Two accessions (AHyxx and Abd-0) were randomly chosen to detect intra-accession polymorphism. The seeds of AHyxx were field collected from at least 30 individual plants in Yuexi County, Anhui Province in southeast China [7]. The seeds of Abd-0 were obtained from the ABRC but were originally collected in the UK. Green leaf material was harvested from 10 individual plants for both accessions and total cellular DNA was extracted separately. PCR amplification, purification and sequencing were conducted as described above.

The DNA sequences were aligned initially using MEGA 4.1 [41]. After alignment, the sequences were edited manually. Gaps were positioned to minimize nucleotide mismatches. The 3'- and 5'- flanking regions of the protein-coding sequences were trimmed off and only the intergenic spacer or intron regions were retained for further analysis.

The nucleotide sequences of 11 fragments from each accession were concatenated sequence by sequence to form a combined sequence set. The combined sequence sets formed a combined data matrix of 77 × 8978 nucleotides.

The sequences of these fragments have been submitted to the GenBank [42], and their accession numbers are listed in Table 4.

### Population genetics and phylogenetic analyses

Polymorphism analyses were conducted for both intra- and inter-specific comparison using DnaSP4.50 [43]. Nucleotide variation was estimated as nucleotide diversity (π) [44]. The number of haplotypes was denoted by *h *and haplotype diversity was denoted by *Hd*. Some statistical methods, such as Tajima's D test [45] and Fu and Li's D*, D, F* and F test [46], were conducted to test the neutral mutation hypothesis. The *A. arenosa *orthologs were used as the out-group sequences when needed.

Phylogenetic analyses were performed on the combined data matrix. Both neighbor joining (NJ) and maximum parsimony (MP) methods were used. The NJ tree was constructed using MEGA 4.1, and Maximum Composite Likelihood distance [47] was used and gaps were treated as a complete deletion. The analysis was done with 1000 bootstrap replicates of the data. The MP tree was constructed using PAUP*4.0 [48]. Bootstrap values were calculated from 1000 replicates with a heuristic search strategy. Gaps in the alignments of the sequences were treated as missing data.

To visualize the relationship among haplotypes, a network was constructed as described by [49] using NETWORK version 4.2.0.1. A default weight of 10 was applied to each site. 'Epsilon = 0' was chosen for constructing the network for the 77 *A. thaliana *accessions using the median joining algorithm.

### Estimated divergence time and demographic analysis

To estimate the divergence time for the *A. thaliana *accessions, the homologous sequences of *A. arenosa *were used as a reference. The divergence time between *A. arenosa *and *A. thaliana *was estimated as 5.1-5.4 million years ago (mya) [50]. In another study, Clauss and Koch estimated a divergence time between these two species as 3.0~5.8 mya [33]. Here we adopted a divergence time of 5 mya to simplify the calculations [51]. MEGA 4.1 was used to calculate the number of base substitutions per site from averaging over all sequence pairs (K) [52] between *A. arenosa *and the 77 *A. thaliana *accessions. Standard error estimates were obtained by a bootstrap procedure with 1000 replicates. All positions containing gaps and missing data were eliminated from the dataset (complete deletion option). The substitution rate per nucleotide site per year (*μ*) was calculated as:(1)

where *T *is the divergence time between *A. arenosa *and *A. thaliana*. The K value between two *A. thaliana *accessions (or groups) was calculated using MEGA 4.1 as mentioned above. The values for the divergence time of the two *A. thaliana *accessions (or accession groups), *T*, was calculated as:(2)

To test for evidence of demographic population growth, a mismatch distribution analysis [53-55] was conducted by using Arlequin 3.11 [56]. Parametric bootstrapping with 1000 replicates was used to evaluate the sum of squared deviations (SSD) between observed and expected mismatch distribution, the raggedness index of Harpending (HRag) [57], the mode of the mismatch distribution (τ), and the upper and lower 95% confidence limits around the estimate of *τ*. For estimating the expansion time *t*, we adopted:(3)

where *t *is the expansion time in number of generations, *τ *is the mode of the mismatch distribution, and *u *is the mutation rate per generation for the entire DNA sequence [54,55]. The *u *was calculated as:(4)

where *m*_T _is the number of the nucleotides of the entire DNA sequence, *μ *is the substitution rate per nucleotide site per year, and *g *is the generation time in years.

## Authors' contributions

PY collected some samples, conducted PCR amplification, DNA sequencing, and data analysis, and wrote the first draft of the manuscript. JK collected some samples, and helped in analyzing the data. FH collected most of the samples and helped in analyzing the data. L-JQ and HG designed the study, and HG conceived of the study and revised the manuscript. All authors read and approved the final manuscript.
